# Expectations, experiences and attitudes of patients and primary care health professionals regarding online psychotherapeutic interventions for depression: protocol for a qualitative study

**DOI:** 10.1186/1471-244X-13-64

**Published:** 2013-02-20

**Authors:** Jesús Montero-Marín, José Miguel Carrasco, Miquel Roca, Antoni Serrano-Blanco, Margalida Gili, Fermin Mayoral, Juan V Luciano, Yolanda Lopez-del-Hoyo, Barbara Olivan, Francisco Collazo, Ricardo Araya, Rosa Baños, Cristina Botella, Javier García-Campayo

**Affiliations:** 1 Department of Psychiatry, University of Zaragoza, Zaragoza, Spain; 2 Faculty of Health and Sports, University of Zaragoza, Huesca, Spain; 3 Research Unit, Sociedad Española de Reumatología, Madrid, Spain; 4 Institut Universitari d’Investigació en Ciències de la Salut (IUNICS), University of Balearic Islands, Palma de Mallorca, Spain; 5 Parc Sanitari Sant Joan de Déu, and Fundación Sant Joan de Déu, Sant Boi de Llobregat, Barcelona, Spain; 6 Psychiatric Service, University Hospital Carlos Haya, Malaga, Spain; 7 Departamento de Psicología y Sociología, Universidad de Zaragoza, Zaragoza, Spain; 8 Servei de Psiquiatria, Hospital Universitari Vall d’Hebron, Barcelona, Spain; 9 Academic Unit of Psychiatry, School of Social and Community Medicine, University of Bristol, Bristol, UK; 10 University of Valencia, Valencia, Spain; 11 Jaume I University, Castellón, Spain; 12 CIBER Pathophysiology of Obesity and Nutrition (CB06/03), Carlos III Institute of Health, Madrid, Spain; 13 Psychiatry Service, Miguel Servet Hospital, Avda Gomez Laguna 52, 4D 50.009, Zaragoza, Spain

**Keywords:** Depression, Online psychotherapy, Internet-based treatment, Protocol, Qualitative study

## Abstract

**Background:**

In the year 2020, depression will cause the second highest amount of disability worldwide. One quarter of the population will suffer from depression symptoms at some point in their lives. Mental health services in Western countries are overburdened. Therefore, cost-effective interventions that do not involve mental health services, such as online psychotherapy programs, have been proposed. These programs demonstrate satisfactory outcomes, but the completion rate for patients is low. Health professionals’ attitudes towards this type of psychotherapy are more negative than the attitudes of depressed patients themselves. The aim of this study is to describe the profile of depressed patients who would benefit most from online psychotherapy and to identify expectations, experiences, and attitudes about online psychotherapy among both patients and health professionals that can facilitate or hinder its effects.

**Methods:**

A parallel qualitative design will be used in a randomised controlled trial on the efficiency of online psychotherapeutic treatment for depression. Through interviews and focus groups, the experiences of treated patients, their reasons for abandoning the program, the expectations of untreated patients, and the attitudes of health professionals will be examined. Questions will be asked about training in new technologies, opinions of online psychotherapy, adjustment to therapy within the daily routine, the virtual and anonymous relationship with the therapist, the process of online communication, information necessary to make progress in therapy, process of working with the program, motivations and attitudes about treatment, expected consequences, normalisation of this type of therapy in primary care, changes in the physician-patient relationship, and resources and risks. A thematic content analysis from the grounded theory for interviews and an analysis of the discursive positions of participants based on the sociological model for focus groups will be performed.

**Discussion:**

Knowledge of the expectations, experiences, and attitudes of both patients and medical personnel regarding online interventions for depression can facilitate the implementation of this new psychotherapeutic tool. This qualitative investigation will provide thorough knowledge of the perceptions, beliefs, and values of patients and clinicians, which will be very useful for understanding how to implement this intervention method for depression.

## Background

Reports from the World Health Organization say that depression will cause the second highest level of disability worldwide by the year 2020 [1]. It is also known that 25% of the population will experience depression symptoms at some point in their lives [2]. Studies confirm that 25-35% of patients visiting primary care suffer from a psychiatric disorder and that more that 80% of these cases are minor psychiatric disorders, mainly depression and anxiety [3]. It is known that family physicians only refer 5-10% of the psychiatric pathologies that they detect to mental health services [4]. Despite this low referral rate, mental health services in Western countries are overburdened. Given the high prevalence of minor psychiatric disorders, health officials worldwide assume that it is not possible to count on the availability of mental health professionals or economic resources to meet this need, and the situation will become even more unfavourable in the near future [5]. For this reason, cost-effective alternatives are being proposed for the treatment of minor psychiatric disorders in general, and depression in particular, that do not involve (or only minimally involve) mental health services. The therapeutic solutions that are most frequently investigated are brief psychotherapies that can be administrated from a personal computer, such as conflict resolution therapy [6], bibliotherapy [7], self-help programs [8] and computer-assisted psychotherapy programs [9].

The term “computer-assisted psychotherapy” is used to refer to any psychotherapy program (all programs currently available use cognitive-behavioural therapy) that uses the patient’s responses to perform some type of decision-making about treatment [10]. This term excludes videoconferences and self-help programs that involve exclusively bibliotherapy, chats, or support groups, among other approaches. Computer programs decrease the work of psychotherapists by more than 80% but do not eliminate it completely. In fact, it has been shown that treatment programs with no human intervention are associated with a higher frequency of dropouts, so their effectiveness is lower. Patients tend to access therapy from a home computer and usually complete short sessions, approximately 20 minutes long, at least once a week for 3–6 months [10]. Research has assessed the effectiveness of computer-assisted psychotherapy for treating psychiatric disorders as varied as anxiety, depressive disorders, alcohol abuse, and psychosomatic illnesses [11-13]. Recent research on the cost-effectiveness of computer-assisted psychotherapy has yielded very satisfactory results [14,15].

In the specific case of depression, studies show that computer-assisted psychotherapy is effective for the treatment of light and moderate depression [9]. This result has led the National Institute for Health and Clinical Excellence of the British National Health Service to support the widespread use of a computer-assisted psychotherapy program (“Beating the Blues”) in the treatment of depression [10]. Other studies show that computer-assisted psychotherapy is very effective, indicating that the program could be viable not only at the primary care level but also in the mental health services context. Computer-assisted psychotherapy could be recommended as a first treatment step for self-help in treating depression and anxiety before visiting a psychiatrist or psychologist [16]. Recent studies have evaluated computer-assisted psychotherapy programs, such as “Blues Begone”, in which there is no psychotherapist present and the program can be administered completely by the patient [17]. The effectiveness of this type of program is also very high, and its use has been evaluated through naturalistic studies and randomised trials [17,18]. The effect size is 0.5 (Cohen’s *d*) when analysed by intention to treat and 1 (Cohen’s *d*) when only the patients who complete the program are analysed. In addition, effectiveness is maintained 6 months after finishing the therapy program [18]. Other computer-assisted intervention models are being tested in which treatment is delivered by a therapist in real time via the Internet, and the results of these tests are positive in terms of effectiveness and acceptability [19].

This type of therapy produces a very positive expectation in patients and a high degree of satisfaction [20,21]. However, there are some limitations to its systematic use. The completion rate for patients in clinical trials is 56%. In most cases, patients withdraw for personal reasons, not because of problems with the technology or the social environment. Interestingly, the attitudes of professionals towards this type of psychotherapy are more negative than the attitudes of patients themselves [20,21]. There are few previous studies on the acceptability of computer-based psychotherapy, particularly in the primary care setting [20].

### Objectives

The first objective of this research is to describe the profile of depressed patients who would benefit the most from online-assisted psychotherapy. The second objective is to identify expectations, experiences, and attitudes among both patients and health professionals that may serve as barriers to or facilitators of online-assisted psychotherapy by considering the types of information that they require throughout the therapeutic process for adequate intervention development.

## Methods

### Design

A qualitative study in the context of a randomised controlled trial on the effectiveness of an online psychotherapeutic intervention for depression is proposed. Through in-depth interviews [22], first, an attempt will be made to understand the experiences of depressed patients with online psychotherapy interventions, the reasons why people decided to end treatment, and the expectations of those who are not treated (considered separately to avoid the possible additional therapeutic effect of the interviews). Second, the research will aim to understand the attitudes and experiences of family physicians, department heads, and managers regarding the implementation of this type of therapy. Focus groups [22] will also be formed to explore dynamic interactions in light of cultural characteristics and concrete values that can be the basis for participants’ views and preferences. In this way, qualitative information will be obtained from a collective and group vision that only emerges from individual interviews with some difficulty [23-25]. This methodological triangulation will increase the consistency and rigor of the study by combining multiple techniques. By placing emphasis on different aspects or perspectives of analysis, these techniques complement and balance each other [26]. The project has been approved by the Regional Ethics Committee of Aragón (Spain).

### Participants

The study will be performed in the Spanish autonomous communities of Andalucía, Aragón, and Baleares. The patients will be recruited during visits to a primary care physician, and diagnosis will be established using the MINI Neuropsychiatric Interview in Spanish [27]. All participants chosen will report depressive symptoms as listed in the ICD-10 criteria, with Beck Depression Inventory (BDI) scores indicating depression of low severity (i.e., scores of 14–19) [28]. Family physicians, department heads, and managers will be chosen for the implementation of the program prepared for the randomised controlled trial. The patients and health professional participants will be selected while considering the variables that maintain homogeneity and discursive heterogeneity regarding the objectives of the study, such as age, gender, residential setting, affinity for technology, and opinion of other types of interventions or therapies. The department heads and managers, as a minority group, will be chosen only based on whether their workplace is rural or urban. When recruiting patients, we will exclude those patients from the study who are younger than 18 or older than 65 years old, immigrants with language difficulties that prevent them from completing the program, affected by serious psychiatric disorders (e.g., psychosis, dementia), or demonstrate clear technophobia during the interview. Through purposive sampling, an attempt will be made to capture rich and varied information in agreement with the goals of the study [29], thus optimising the representation of participants’ opinions.

A preliminary analysis will allow us to progress though the interviews in an iterative manner until the data are saturated; that is, until the new information becomes redundant and provides no new perspectives. We hope that this will occur after approximately 10 interviews in each of the four groups (depressed patients who completed online psychotherapy; depressed patients who decided to discontinue online psychotherapy treatment; depressed patients who did not receive treatment online; and family physicians, department heads, and managers). After the interviews, four focus groups will be conducted, each consisting of between eight and ten subjects. An attempt will be made to ensure the adequate representation of the stratification variables considered but with intergroup heterogeneity and intragroup homogeneity to allow the groups to be formed appropriately [24,30]. The necessary information to establish the appropriate stratifications will be collected before beginning the interviews and focus groups.

### Data collection

The researchers involved in the study will receive training in qualitative social research procedures, in-depth interviews, and focus groups [22]. Topics to be addressed in both the individual interviews and focus groups will be discussed by the team of researchers and will guide the interviewers and moderators in the same direction. In-depth interviews will be carried out by a single interviewer, who will indirectly raise the objectives of the study, questioning interviewees about topics in an open and progressive way. The interviewer will also explain the need for recording the session (only audio) and will take notes about non-verbal language elements. The focus groups will be moderated by an interviewer, and another person from the research team may be present as an observer. The role of the moderator will be to indirectly explain the objectives of the study, introduce the topics of interest, and direct the group dynamics to encourage dialogue and participation. The function of the observer is limited to collecting field notes to provide additional information to the verbal data obtained, such as information related to non-verbal language, responses to the moderator’s interventions, and contextual aspects. The indirect introduction of the objectives will enable us to analyse the participants’ natural approach to the issues of interest.

We will seek to achieve optimum standardisation in the sessions with a specification guide with open and flexible content (Table 1) while allowing the inclusion of issues introduced by the participants [31]. The basic topics to explore, pre-selected by a panel of experts (psychiatrists, psychologists, and sociologists with experience in applying new mental health technologies), will include key issues such as training in new technologies, opinions about therapy in general and about the use of computer-assisted psychotherapy [20], accommodation of therapy into the daily routine, the possibility of developing a virtual and anonymous relationship with the therapist, the process of online communication of thoughts and emotions, re-reading and revising the written material describing thoughts and emotions [32], information needed to adequately address the online therapeutic process [33], material conditions for practice, program content and personal preferences, identification with and applicability of the program, professional support and adherence, experiences of symptomatic changes and their possible relationship with the program [34], continual engagement with the program, and facilitators, barriers, and expected consequences that may result from this type of intervention [35]. Specific topics will be added to understand how to offer this therapy in the primary care context and the modification of the doctor-patient relationship that may result. When interviewing the department heads and managers, resources and risks will be addressed.

**Table 1:** Topic list

**Areas**	**Issues**
**Computer aspects**	-Information technology skills
	-Material resources
	-Routine use
**Expectations**	-Therapeutic expectations
	-Online vs. in person therapy
**Experiences**	-Identification with the program
	-Barriers and facilitators
	-Changes in symptoms
**Attitudes**	-Expressed possibilities
	-Anonymous and virtual relationship
	-Reflectivity of responses
**Information**	-Information and preferences
**Staff**	-Normalisation in primary care
	-Professional support
**Improvements**	-Interface
	-Program
	-Adherence

We will ensure the confidentiality and anonymous nature of the study for potential participants. All of the sessions will be audio taped with the consent of the participating subjects, and the material will be transcribed verbatim. Both the interviews and the focus groups will last approximately 60–90 minutes. The two researchers responsible for the focus groups will meet after each session to clarify and exchange views and field notes. This information will be analysed with data from the audio recordings and notes [31].

### Data analysis

The body of text (verbatim) will consist of recordings transcribed literally, supplemented by notes from observations and comments from interviewers/moderators and collaborators. The interviewers/moderators will verify the transcripts to guarantee the accuracy of the data. Final validation will be performed by inviting the participants to read and discuss the transcribed content [36].

The preliminary analysis will begin after the first interviews and progress through the interviews iteratively to confirm or discount the topics found [37]. The transcriptions and notes will be reread and analysed by two independent social researchers who are experts in qualitative content analysis, using a vertical, interpretive, emergent, and non-frequency-based approach [22,38]. Through thematic analysis, supported by the initial guide for content specification as the first framework for approximation and using the constant comparative method from fundamental theory, key units of meaning will be identified that enable us to deconstruct every sentence [39]. The information contained in the generated text segments will be compared and grouped through open coding until a common conceptual denomination for all segments of text that share the same thematic unit is reached [40]. Provisional interpretations will be made to highlight the characteristics and relationships of the emerging codes generated, which will form new categories as a result of their gradual fusion [39-41]. These broader categories, defined in an exclusive manner based on agreement among the researchers, will make the conceptual structure denser until a parsimonious solution can be reached. Cases that cannot be classified will be actively sought out during the analysis, and the emerging categories will be redefined in response to these cases [42,43].

The information gathered from focus groups will be analysed using the model of sociological analysis [44,45]. In this technique, the reading and organisation of data are not based on fragmentation of the text but on the interpretation of the different discursive positions among the participants and the identification of the explanatory axes of their interventions [46]. The themes and patterns will be identified and coded by two independent researchers, taking into account the context in which the interventions took place. Each step towards configuring the potential explanatory axes will require an iterative reading to validate and confirm the interpretation of new findings, ensuring the credibility of the process by reviewing the data again from the beginning [47]. At first, the material from different groups will be analysed separately (intra-group analysis), but later, it will be regrouped and pooled to compare the relevant themes (inter-group analysis). The precision of the analysis will be increased by highlighting the consistent results in all groups, and special attention will be given to “sensitive moments” in the interaction as indicators of important discussions. The whole process will be triangulated between the two researchers, and differences will be resolved through discussion.

To manage the data, we will use the analysis program MAXQDA 2007. The results of the thematic content analysis of the interviews and the interpretation of the main explanatory axes in the focus groups will be presented alongside the empirical references in the text, selecting the most representative verbatim segments for use as examples. The researchers will develop a list of concepts identified in the interviews and focus groups and create conceptual diagrams that increase understanding and comparability. The final coding framework will be discussed with the main researcher and therapy coordinator so that all stakeholders agree.

## Discussion

Online computer-assisted psychotherapy seems to be an effective and cost-effective approach to treating mild to moderate depression [14,16-18]. In fact, referring patients to computer-assisted psychotherapy after consultation in primary care could relieve some of the burden on mental health services, which are presently unable to meet the demand for mental health care [5]. Knowledge of the expectations, experiences, and attitudes of those involved in the therapeutic process is very important if this new form of treatment is to be implemented successfully. To date, research on these issues has been restricted to the perspective of the patient [32-35] without adequately addressing the perspectives of health officials who would be involved in recommending this type of intervention. Interestingly, these professionals show greater resistance to the use of this kind of intervention than the patients themselves do. The majority of patients who discontinue computer-assisted psychotherapy treatment do so for personal reasons and not because of problems with the technology or the social environment [20,21]. The changes introduced to clinical practice and the doctor-patient relationship by computer-assisted psychotherapy make it important to identify the profile of patients who could benefit most from this therapeutic approach. In addition, it is very important to understand the difficulties that may arise during the therapeutic process to address them and to identify facilitators for completing the treatment.

Qualitative research methods provide a thorough understanding of the perceptions, beliefs, and values of the people being studied, and they are very useful in the health field [48,49]. These methods provide the opportunity to explore the points of view of both patients and medical personnel involved in the therapeutic process, thus helping researchers to understand them [29]. This study does not quantify the hypothetical positive or negative aspects of online therapeutic intervention or the correlations between opinions and other types of variables [25,50]. Through this research, an attempt is made to understand the values and experiences of the participants in the context of the study, which are valid to the extent that they contribute to the knowledge and understanding of the therapeutic reality of this type of intervention.

## Competing interests

The authors declare that they have no competing interests.

## Authors’ contribution

JM-M, JMC, ASB, MR, MG, RB, CB, RA and JG-C conceptualised the study. JM-M wrote the manuscript and all authors participated in critically revising for important intellectual content and have given final approval of the version to be published.

## Authors’ information

Jesús Montero-Marín, Javier García-Campayo, Miquel Roca, Antoni Serrano-Blanco, Margalida Gili, Fermín Mayoral, Juan V. Luciano, Yolanda López del Hoyo and Bárbara Oliván belong to the REDIAPP (Research Network on Preventative Activities and Health Promotion, RD06/0018/0017).

## Pre-publication history

The pre-publication history for this paper can be accessed here:

http://www.biomedcentral.com/1471-244X/13/64/prepub
